# Preparation of Preformed Iodixanol Gradients

**DOI:** 10.1100/tsw.2002.285

**Published:** 2002-05-16

**Authors:** John Graham

**Affiliations:** School of Biomolecular Sciences, Liverpool John Moores University, UK

**Keywords:** OptiPrep^TM^, iodixanol, discontinuous gradients, continuous gradients, underlayering, diffusion, freeze-thawing, Gradient Master, two-chamber gradient maker, buoyant density gradients, rate-zonal gradients

## Abstract

The Protocol Article describes the strategies for the formation of preformed discontinuous and continuous density gradients. Most of the techniques can be applied to the use of any gradient solute but some of the detailed recommendations on continuous gradients only apply to iodixanol gradients because of variations in viscosity and rates of diffusion between different solute types. Operational variations that require consideration when dealing with specific types of biological particle are also dealt with at the end of this Protocol Article.

**METHOD TYPE:** extraction, isolation, purification and separation

**SUB METHOD TYPE:** centrifugation

## DISCONTINUOUS GRADIENTS

Discontinuous gradients are best produced by underlayering, using a syringe attached to a metal filling cannula (i.d. 0.7–1.0 mm) or to a piece of nonflexible (Teflon) tubing, low-density end first (Fig. 1).

Overlayering in which the gradient is constructed dense end first is an alternative. Layering can be carried out using a syringe and cannula, a pipette, or a Pasteur pipette. Always keep the tip of the cannula or pipette against the wall of the tube and close to the meniscus of the previous layer.

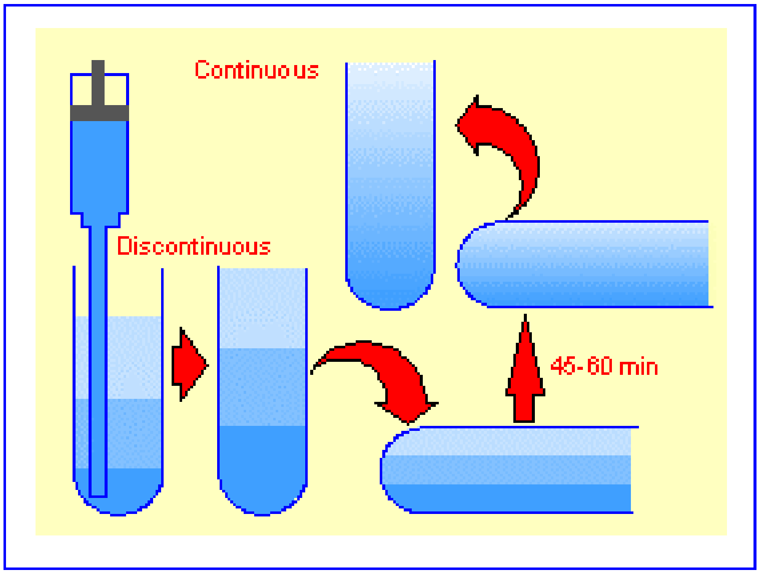

**FIGURE 1**. Formation of preformed discontinuous and continuous gradients (by diffusion).

## CONTINUOUS GRADIENTS

Continuous gradients can be produced in one of four ways.

### 1. From Discontinuous Gradients

If these are allowed to stand for 3–4 h at room temperature or at 4°C for 12 h, diffusion across the interfaces will form a continuous gradient. The time required for the gradient to become linear will depend on the vertical height of each layer, the cross-sectional area of the tube, and the viscosity of the layers. Diffusion will occur more rapidly if the cross-sectional area of the interfaces is increased and the vertical height of the layers decreased by laying the capped tube gently on its side (Fig. 1). For many cell fractionations that are run at room temperature, the cell suspension can be included in one or more of the layers.

The shape of the gradient can be modified by using different volumes for each step and the method provides gradients with highly reproducible density profiles.

### 2. Using a Standard Two-Chamber Gradient Maker

The preferred method is to have the low-density medium in the mixing chamber so the tip of the delivery tube needs to be placed at the bottom of the centrifuge tube (i.e., the gradient is delivered low-density end first). In the alternative strategy in which the high-density solution is placed in the mixing chamber, the tip of the delivery tube needs to be placed against the wall of the centrifuge tube close to its top. The incoming gradient is allowed to flow in a steady stream down the wall of the tube. A more satisfactory method is to deliver the gradient through the Labconco Auto Densi-flow device. The delivery head automatically moves upwards with the rising meniscus of the gradient.

Ideally, for particles that are to be separated at 0–4°C, the gradient should be prepared in a cold cabinet or stored for at least 1 h at this temperature before use.

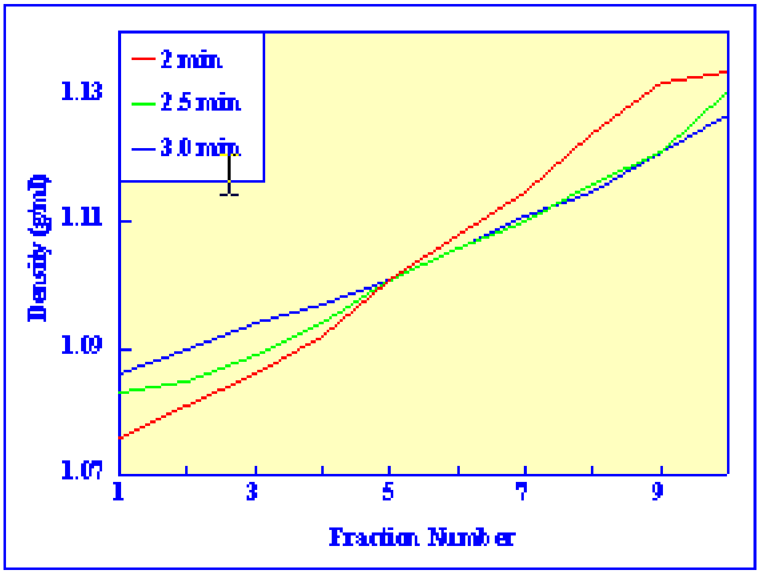

**FIGURE 2**. Formation of continuous gradients in Gradient Master from 5 ml each of 10 and 30% iodixanol (osmotic balancer: 0.25 M sucrose) at 80° at 20 rpm. Effect of time.

Linear gradients are produced if the cross-sectional area of the two chambers is identical; convex or concave gradients are produced if the mixing chamber has a smaller or larger cross-sectional area, respectively.

## 3. Using the Gradient Master

More reproducible continuous gradients are obtained using a Gradient Master™ (or Gradient Mate™) in which the low- and high-density solutions, layered in the centrifuge tube, produce a continuous gradient by controlled mixing. The tubes are rotated at a preset angle (to increase the cross-sectional area of the interface) and speed for about 2 min. The density profiles are highly reproducible. Some examples are given in Fig. 2.

## 4. Freeze-Thawing

The final manner in which continuous gradients can be produced is by freezing a solution of uniform density for approx. 30 min at -20°C and thawing for 30–60 min at room temperature. These times are for tubes of relatively small volume (approx. 5 ml). The freeze-thaw cycles can then be repeated, this modulates the density profile of the gradient (Fig. 3). Generally as the number of freeze-thaw cycles increases the gradient becomes markedly less dense at the top. The method can produce gradients that are more-or-less linear. Because the shape of the gradient depends on the rate of freezing and thawing and the number of freeze-thaw cycles and the volume of the tube, the precise conditions required to generate a certain density profile need to be worked out for a particular laboratory. Under well-controlled conditions however the profiles are quite reproducible.

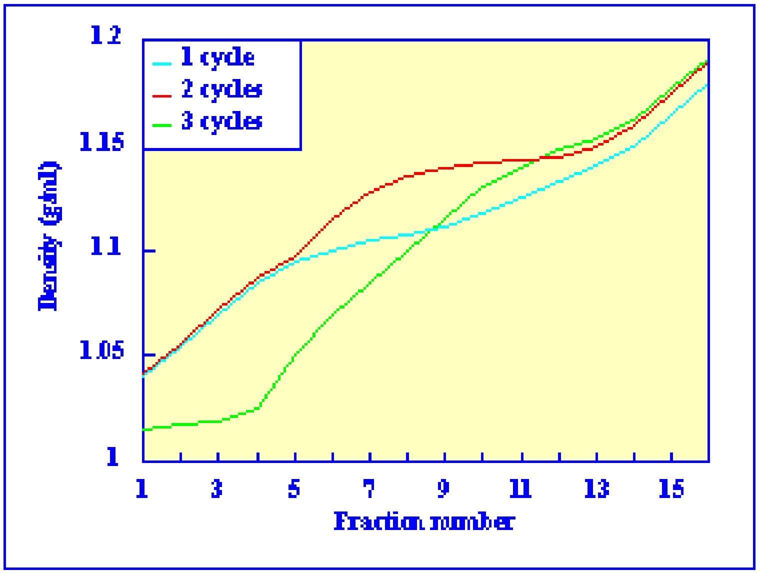

**FIGURE 3**. Density gradient profiles produced by freeze thawing of a 20% iodixanol solution in 5-ml tubes. Effect of number of freeze-thaw cycles.

## SAMPLE HANDLING

### Buoyant Density Separations

The sample can be placed on top, at the bottom, or as part of one of the layers of a discontinuous gradient. It may also be made part of one or more layers of a discontinuous gradient prior to the formation of a continuous gradient by diffusion at room temperature (so long as this takes no more than approx. 45 min). This is clearly not an option for particles that need to be maintained at 0–4°C, nor is incorporation into one or more layers of a discontinuous gradient prior to the formation of a continuous gradient by overnight diffusion at 0–4°C a realistic option for subcellular particles.

The sample may be incorporated into one or both of the layers used in a Gradient Master; this is satisfactory for any particle so long as the gradient is prepared at an appropriate temperature. Continuous gradients formed using a two-chamber device or by freeze thawing must either be top- or bottom-loaded with the sample after gradient formation. This option is indeed available for all continuous gradients.

### Rate-Zonal Separations

Routinely the sample is placed on top of a continuous gradient (separation according to rate of sedimentation), although there is no reason why bottom loading cannot be used (separation according to rate of flotation). Discontinuous gradients tend not to be used for this procedure.

## CHOICE OF ROTORS

Preformed gradients for cell fractionation and for cell nuclei are always carried out in a swinging-bucket rotor. Such gradients for smaller particles are also routinely run in swinging-bucket rotors but vertical rotors are a more efficient option, because of their shorter sedimentation path length. It is also permissible for gradients to be run in fixed-angle rotors, particularly for preparative purposes. Since all modern ultracentrifuges have slow acceleration and deceleration modes to allow smooth reorientation of the gradient in a vertical or fixed-angle rotor tube, the absolute requirement to use a swinging-bucket rotor for gradient work has disappeared.

## ACKNOWLEDGEMENTS

The author and TheScientificWorld wish to thank Axis-Shield PoC, AS, Oslo, Norway for their kind permission to adapt OptiPrep™ Application Sheets C2, M2, and S3 in the preparation of this Protocol Article.
